# Validation of a Monte Carlo Modelling Based Dosimetry of Extraoral Photobiomodulation

**DOI:** 10.3390/diagnostics11122207

**Published:** 2021-11-26

**Authors:** Anna N. Yaroslavsky, Amy F. Juliano, Ather Adnan, Wayne J. Selting, Tyler W. Iorizzo, James D. Carroll, Stephen T. Sonis, Christine N. Duncan, Wendy B. London, Nathaniel S. Treister

**Affiliations:** 1 Advanced Biophotonics Laboratory, Department of Physics and Applied Physics, University of Massachusetts Lowell, Lowell, MA 01854, USA; tyler_iorizzo@student.uml.edu; 2 Department of Dermatology, Massachusetts General Hospital, Boston, MA 02114, USA; 3 Department of Radiology, Massachusetts Eye and Ear, Harvard Medical School, Boston, MA 02114, USA; Amy_Juliano@meei.harvard.edu; 4 Texas A&M Health Science Center, College of Medicine, Houston, TX 77030, USA; aadnan@exchange.tamu.edu; 5 Department of Surgical Science and Integrated Diagnostics, University of Genoa, 16123 Genoa, Italy; wselting@aol.com; 6 THOR Photomedicine, Chesham HP5 1LF, UK; james.carroll@thorlaser.com; 7 Department of Surgery, Division of Oral Medicine and Dentistry, Brigham and Women’s Hospital, Boston, MA 02114, USA; ssonis@biomodels.com (S.T.S.); ntreister@bwh.harvard.edu (N.S.T.); 8 Department of Oral Medicine, Infection and Immunity, Harvard School of Dental Medicine, Boston, MA 02114, USA; 9 Biomodels LLC, Waltham, MA 02451, USA; 10 Department of Pediatrics, Dana-Farber/Boston Children’s Cancer and Blood Disorders Center, Harvard Medical School, Boston, MA 02114, USA; christine_duncan@dfci.harvard.edu (C.N.D.); wendy.london@childrens.harvard.edu (W.B.L.)

**Keywords:** low-level light therapy, Monte Carlo method, in vivo dosimetry, mucositis

## Abstract

An in vivo validation study was performed to confirm the accuracy of extraoral photobiomodulation therapy (PBMT) dosimetry determined by modelling. The Monte Carlo technique was utilized to calculate the fluence rate and absorbed power of light delivered through multi-layered tissue. Optical properties used during Monte Carlo simulations were taken from the literature. Morphological data of four study volunteers were acquired using magnetic resonance imaging (MRI) scans. Light emitting diode (LED) coupled to a power meter were utilized to measure transmitted power through each volunteer’s cheek, in vivo. The transmitted power determined by Monte Carlo modelling was compared to the in vivo measurements to determine the accuracy of the simulations. Experimental and simulation results were in good agreement for all four subjects. The difference between the mean values of the measured transmission was within 12% from the respective transmission obtained using Monte Carlo simulations. The results of the study indicate that Monte Carlo modelling is a robust and reliable method for light dosimetry.

## 1. Introduction

Among patients undergoing hematopoietic cell transplantation (HCT), oral mucositis (OM) is a common and painful side-effect, characterized by mucosal inflammation and ulceration that can result in the inability to eat, drink, and swallow, and can lead to upper airway compromise [1,2,3,4]. Photobiomodulation therapy (PBMT) uses low energy light to evoke anti-inflammatory, analgesic, and other therapeutic biological responses. It has been proven safe and effective for preventing and treating OM in children and adults [5,6,7]. Most PBMT treatment protocols for OM utilize an intraoral approach, requiring the instrument to be held inside the patient’s oral cavity and administered in a spot-by-spot manner directly to the mucosa [8]. This approach can be lengthy, uncomfortable for patients, and logistically challenging. Moreover, it limits PBMT application to the distal oral mucosa.

Extraoral delivery of PBMT using LED arrays ensures simpler application and the ability to treat the oral cavity, oropharynx, and upper oesophagus with a broader treatment field [9]. However, extraoral delivery requires transmission of photons through the external orofacial tissue layers such as skin, fat, and muscle before reaching the inner mucosal lining, attenuating the dose delivered and requiring additional dosimetric considerations. While several small studies describing extraoral PBMT for prevention of OM have been reported, none include a rationale for the selection of treatment parameters used [10]. Monte Carlo simulations could be used to determine the appropriate parameters to deliver the target dose to the mucosa lining. However, the validity of the Monte Carlo simulations to accurately predict the dosimetry of extraoral PBMT treatments must be established prior to the development of treatment protocols for extraoral PBMT. The objective of this study was to examine the accuracy of Monte Carlo simulations to predict the fluence rate and absorbed power distribution in tissue.

## 2. Materials and Methods

### 2.1. Study Volunteers

This study was approved by the Institutional Review Boards of the Dana-Farber/Harvard Cancer Centre and Massachusetts Eye and Ear (protocol # 16-164, 19 April 2016). Four adult volunteers participated in this study. Study volunteers included a 25-year-old male with skin type IV, a 43-year-old male with skin type I, a 25-year-old female with skin type II, and a 57-year-old female with skin type VI.

### 2.2. Volunteer MRI and Image Analysis

Images of each volunteer’s face were acquired using an axial T1-weighted MR imaging sequence (Philips, Amsterdam, The Netherlands). A line was drawn on the selected image, simulating the trajectory of light delivered through the cheek during the light transmission measurements. This line was in an oblique orientation relative to the horizontal, and perpendicular to the tangent at the cheek surface, extending from the skin surface to the oral buccal surface. The thickness of each tissue type encountered along this line—skin, fat, and muscle layers—was measured on the image using electronic callipers on a standardized PACS viewing station (Synapse, Fuji, Japan).

### 2.3. LED Light Source

**Figure 1:**
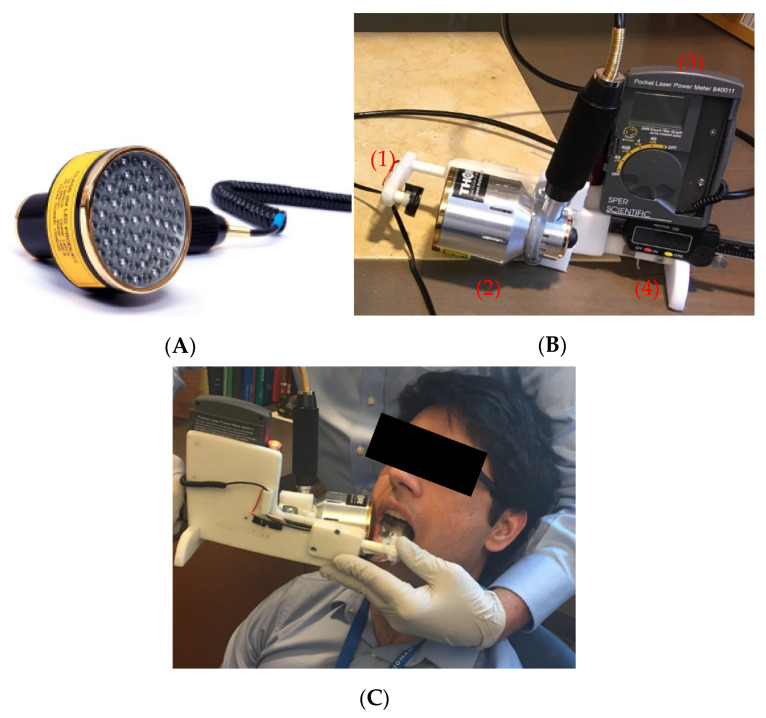
(**A**) LED probe provided by THOR Photomedicine Ltd. (**B**) Device used in validation study: (1) power meter sensor, (2) LED probe light source, (3) SPER Scientific Pocket Laser Power Meter, and (4) calliper for measuring cheek thickness. (**C**) The device used for in vivo transmission measurements. The LED probe was placed on the outside of the volunteer’s right cheek, with the light sensor placed on the surface of the mucosa, intraorally.

The LED probe used for in vivo measurements was provided by THOR Photomedicine Ltd. (Chesham, UK) (Figure 1A). The probe consisted of 69 LEDs that emitted 850 nm light with a beam divergence of 22°. Each LED had a full width half maximum of 45 nm, and an active area of 0.2 cm^2^. The maximum power density of the probe was 28 mW/cm^2^. The probe had an outer diameter of 70 mm; the active area of the probe had a diameter of 63 mm. To test output stability, power measurements of the probe were made over the course of 5 min via a SPER Scientific Pocket Laser Power Meter–840011 (Scottsdale, Maricopa, AZ, USA). Output uniformity over the active area of the probe was also tested. Approximately 26.0 ± 5.82 × 10^−4^ mW/cm^2^ was delivered uniformly over an area of 31.2 cm^2^.

### 2.4. Transmission Measurement Device

The apparatus used for in vivo measurements of light transmission through the volunteers’ cheeks consisted of a light sensor, a power meter, a calliper, and an LED probe described above (Figure 1B). An SPER Scientific Pocket Laser Power Meter (Scottsdale, AZ, USA) was used to measure radiated power. The power meter light sensor (an Si photodiode) was mounted onto the calliper, facing toward the LED probe. The photodetector with a measurement area of 0.2827 cm^2^ was mounted inside a platform with a surface area of 5 cm^2^. The sensor could be moved toward and away from the LED probe. The calliper measured the distance between the light source and sensor.

### 2.5. Transmission Measurements and Analysis

Transmission of 850 nm light through each volunteer’s cheek was measured using the device described above (Figure 1). First, the output of the LED probe was measured as a reference. Then, the LED probe was placed onto the exterior surface of the volunteer’s cheek while the light sensor was placed onto the interior side of the cheek (Figure 1C) and transmitted power through the cheek was measured. Using the reference power measurement (*I*_0_), transmitted power measurement (*I*), and the thickness of the cheek (*x*), the effective attenuation coefficient (*µ*) of the cheek was calculated using Beer’s Law (Equation (1)). This process was repeated 8 times per volunteer. The average effective attenuation coefficient, average cheek thickness, and average reference power measurement was then calculated. Using these values, the average transmitted power through the volunteer’s cheek was calculated using Beer’s law.

(1) $${I=I}_{0}e^{-µx}$$

### 2.6. Monte Carlo Simulations

The Monte Carlo technique, described in detail elsewhere [11], was used to simulate the propagation of 850 nm light through each volunteer’s cheek. In short, fluence rate and absorbed power distributions, as well as the light transmitted through the medium (transmission) and reflected by the medium (reflection) were calculated for a parallel plane, multilayer scattering, and absorbing medium assuming monochromatic incident light. Each layer of the medium was characterized by the following parameters: ($\mu_{ai},\mu_{si},p_{i}\left(\hat{s},\hat{s}{}^{\prime }\right),d_{i},n_{i}$), where *µ_ai_* is the absorption coefficient, *µ_si_* is the scattering coefficient, $p_{i}\left(\hat{s},\hat{s}{}^{\prime }\right)$ is the scattering phase function, *d_i_* is the thickness, and *n_i_* is the refractive index of the respective layer *i*. Spatial and angular distributions of the incident light were assumed to be radially symmetric:

(2) $$S\left(\overset{¯}{r},\hat{s}\right)=A(\hat{s})E\left(\overset{¯}{r}\right),$$

where $A\left(\hat{s}\right)$ is angular distribution of a unit source. Since translational symmetry exists with respect to the layer boundaries, $E\left(\overset{¯}{r}\right)$ is radial distribution of the source.

**Figure 2:**
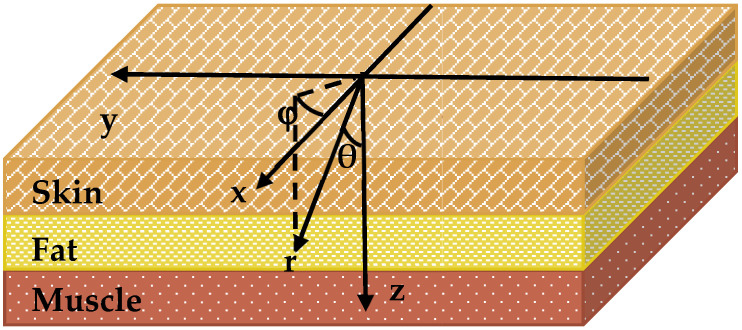
Layer model diagram of facial tissues with coordinate system used in Monte Carlo simulations.

The modelled medium was divided into voxels (*i*, *j*, *k*). If *N* is the number of layers, then $d=\sum_{i=1}^{N}d_{i}$—is the thickness of all medium layers 0≤z≤d. Photons were placed at the origin of the coordinate system and assigned initial weight W_0_. As shown in Figure 2, each photon is characterized by three coordinates $\bar{r}$ (*x*, *y*, *z*) and two angles (θ, φ), which determine its direction of propagation. θ is counted from the positive z-axis and φ—from the positive x-axis in (*x*, *y*) plane. The photon path length,$\;l_{rnd}=-ln(1-\alpha )/\mu_{t}$, and the direction of the photon propagation is determined using the following formula:

(3) $$\alpha =\int_{x_{min}}^{x_{rnd}}p\;\left(x\right)dx$$

where *α*—is a random number uniformly distributed in the interval (0,1), *p*(*x*)—is the function of the probability distribution of the random variable *x*. *x_min_*—is minimal *x* value and *x_rnd_*—is the random *x* value.

After calculating the path length and scattering angle, the algorithm tested whether the photon crossed the tissue boundary. If not, the photon was moved to the point predetermined utilizing Equation (3). If the photon did cross it, Fresnel formulas were used to calculate the probability of its reflection off the respective boundary. If the photon crossed the boundary between layer *i* and *i + 1*, Snell’s law was employed to calculate the new direction of propagation and the remaining pathlength *l’* was calculated as $l{}^{\prime }=l*n_{i}/n_{i+1}$, where *l* is a remaining path length calculated for *i* layer of the medium. Part of the photon’s weight proportional to (1 *−* c), where *c* is albedo of the layer, was counted as an element *Q_ij_* of the matrix that quantifies the distribution of energy absorbed in the medium. The values of the indices (*i*, *j*) were determined using updated photon coordinates. Then, new values of the pathlength and scattering angle were generated, and the procedure was repeated. When photon’s weight was reduced below a certain predetermined value, “Russian roulette” was played [12]. In the case when the photon exited the medium, part of its weight proportional to the transmission coefficient of the medium boundary was counted as an element *Q_Tj_* of the matrix that quantifies transmission. Part of its weight proportion was counted as an element *Q_Rj_* of the matrix quantifying reflectance. The value of index *i* was determined for the updated photon coordinates, using the formula $\sqrt{x^{2}+y^{2}}$.

After repeating the algorithm for a significantly large pre-set number of photons *N_phs_*, Green functions of the medium were calculated using the following formulas:

(4) $$G_{ij}=Q_{ij}/N_{phs}Vij\mu_{aij}W_{0},$$

(5) $$G_{li}=Q_{li}/N_{phs}S_{i}W_{0},$$

where $V_{ij}$—is volume of the voxel *(i*, *j)*, $\mu_{aij}$—absorption coefficient of the medium layer of voxel *(i*, *j)*, *l =* T or R for transmission and reflection, respectively, and $S_{i}$—area of the ring corresponding to the distance $\sqrt{x^{2}+y^{2}}$.

Finally, the spatial and angular distribution of the incident light and green functions (4 and 5) of the medium were utilized to calculate fluence rate distribution $FR\left(\overset{¯}{r}\right)$ and absorbed power distribution $AP\left(\overset{¯}{r}\right)$ within the medium:

(6) $$AP\left(x,y,z\right)=\iint^{\;}G\left(x^{\prime },y^{\prime },z\right)E\left(x-x^{\prime },y-y^{\prime }\right)dx^{\prime }dy^{\prime },$$

(7) $$FR_{l}\left(x,y\right)=\iint^{\;}G_{i}\left(x^{\prime },y^{\prime }\right)E\left(x-x^{\prime },y-y^{\prime }\right)dx^{\prime }dy^{\prime },$$

where *l = T* or *R* for transmission and reflection, respectively, and *x*, *y*, *z* are Cartesian coordinates.

To determine the accuracy of the Monte Carlo simulations, the percent difference between simulated and measured transmitted power was calculated. The optical properties of each tissue were taken from the literature [13,14]. Properties were assigned to each image voxel based on the location of the respective tissue layer (Figure 2). The thickness of each tissue layer was determined using acquired MR images of each study volunteer.

### 2.7. Optical Properties

The optical properties, including absorption coefficients and reduced scattering coefficients used in Monte Carlo simulations were taken from the literature (Table 1). The optical properties of skin were determined in vivo by Tseng et al. (2009) [13]. The optical properties of fat and muscle were measured ex vivo by Simpson et al. (1998) [14]. The anisotropy factors were set to 0.9.

## 3. Results

### 3.1. MRI and Image Analysis

**Figure 3:**
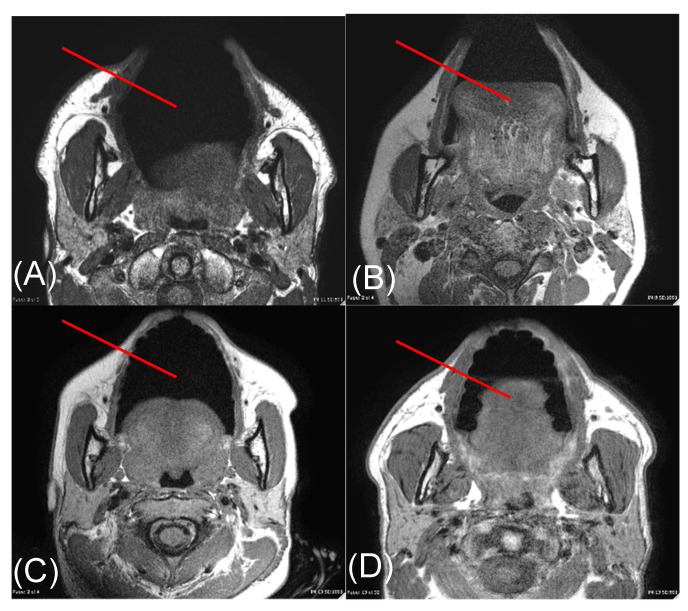
Axial T1-weighted MR images through the face acquired from subjects 1 (**A**), 2 (**B**), 3 (**C**), and 4 (**D**). The red line on each image depicts the trajectory along which a representative light beam travels during transmission measurements. Measurements were made along this red line from the surface of the skin to the surface of the buccal mucosa.

MR images acquired from each volunteer are shown in Figure 3. Tissue layers contained within the right cheek of volunteers were determined to be skin, fat, and muscle (Columns 5–7 of Table 2). Skin thicknesses among volunteers ranged from 1 to 2 mm, fat ranged from 0 to 3 mm, and muscle 3 to 7 mm. Subjects 2 and 3 had the thinnest total cheek width (6 mm), whereas subject 1 had the thickest (12 mm).

### 3.2. Transmission Measurements and Analysis

Average measured transmitted fluence rates were 0.47 mW/cm^2^, 2.14 mW/cm^2^, 2.38 mW/cm^2^, and 1.07 mW/cm^2^ for study subjects 1, 2, 3, and 4, respectively (Table 2). As shown by the measured data, transmitted fluence rate greatly depends on the total cheek thickness but does not significantly depend on the skin type. The measured transmission values from Subject 1 (greatest total cheek thickness) differed from the measured values from Subjects 2 and 3 (smallest total cheek thickness) by 127% and 134%, respectively.

### 3.3. Monte Carlo Simulations

**Figure 4:**
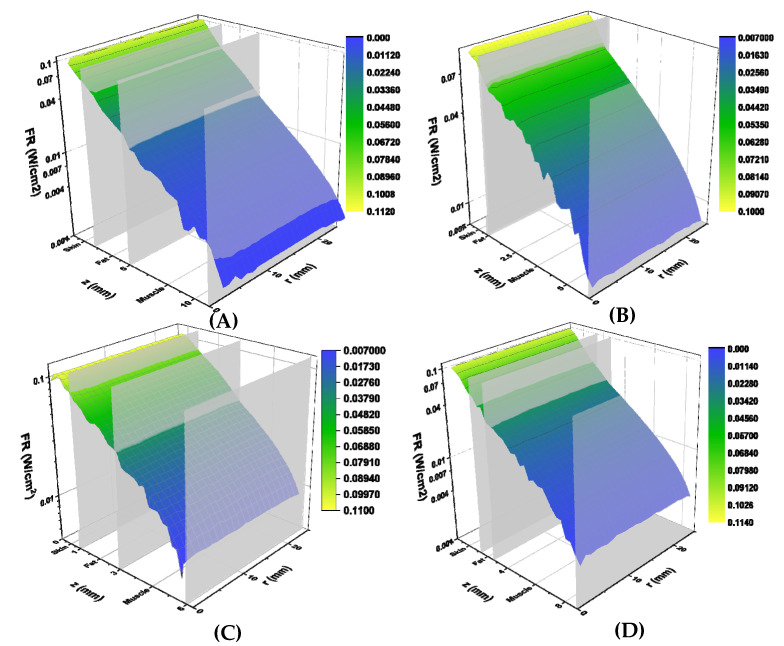
Simulated fluence rates (W/cm^2^) for volunteers 1 (**A**), 2 (**B**), 3 (**C**) and 4 (**D**). Calculated fluence rates are displayed on the vertical axis, tissue depth (mm) is displayed on the z axis, and lateral tissue radius (mm) is displayed on the r axis. Tissue boundaries are labelled on the tissue depth axis. Graphs are colour-coded based on fluence rate, with yellow corresponding to higher fluence and blue to lower fluence rates.

The calculated fluence rates (W/cm^2^) for each study volunteer are shown in Figure 4. Fluence rates are plotted against total tissue depth (z-axis) and lateral tissue dimension (r-axis). Individual tissue layer boundaries are labelled on the z-axis, indicating where each layer ends. Simulated fluence rates decreased exponentially with increasing tissue thickness but did not vary greatly in the lateral direction. Average transmitted fluence rates were determined to be 0.41 mW/cm^2^, 2.12 mW/cm^2^, 2.07 mW/cm^2^, and 0.82 mW/cm^2^ for study subjects 1, 2, 3, and 4, respectively (Table 2).

**Figure 5:**
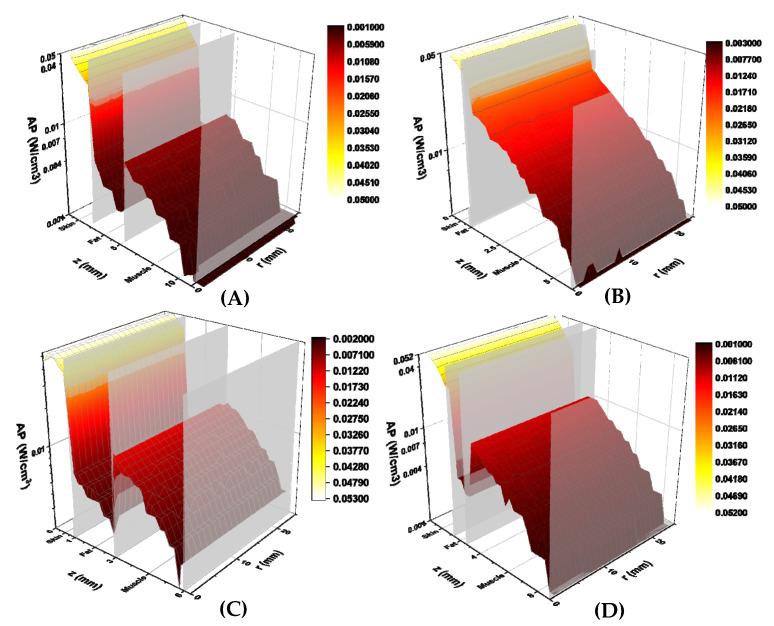
Simulated absorbed power (W/cm^3^) for volunteers 1 (**A**), 2 (**B**), 3 (**C**), and 4 (**D**). Calculated absorbed power are displayed on the vertical axis, tissue depth (mm) is displayed on the z axis, and lateral tissue radius (mm) is displayed on the r axis. Tissue boundaries are labelled on the tissue depth axis. Graphs are colour-coded based on absorbed power, with yellow illustrating higher power and red lower power.

The calculated absorbed power (W/cm^3^) for each study subject is shown in Figure 5. Absorbed power is plotted against total tissue depth (z-axis) and lateral tissue radius (r-axis), similar to the calculated fluence rates. Power levels decreased exponentially with tissue depth and did not change significantly with tissue radius. A sharp decrease in absorbed power was found at the skin-fat tissue boundary in subjects 1, 3, and 4. This was followed by an increase in absorbed power at the fat-muscle tissue boundary. This behaviour may be explained by the lower absorption coefficient of the fat, as compared to both skin and muscle [13,14]. No fat tissue layer was detected in subject 2′s MR images, thus only a slight decrease in absorbed power at the skin-muscle tissue boundary was observed.

With respect to the percent differences between measured and calculated transmission, subject 1 had the greatest percent difference of 12%, whereas subjects 2 and 4 had the smallest percent difference of 1% (Table 2). All study subjects had a positive percent difference, indicating that measured fluence rates were greater than the simulated values among all subjects.

## 4. Discussion

Our model of extraoral PBMT is an important first step in developing an evidence-based extraoral treatment protocol. Currently, PBMT protocols recommended for OM involve intraoral delivery [8]. The treatment parameters surrounding these protocols vary slightly depending on the disease and cancer therapy and even institutional differences, but generally fall within a set range: wavelength of 632.8–660 nm, power density of 24–31.25 mW/cm^2^ for He-Ne lasers and 417–1000 mW/cm^2^ for diode lasers, and a target energy density of 1–6.2 J/cm^2^ [8]. The few reports of extraoral PBMT for OM use a treatment protocol similar to those utilized intraorally [15]. However, the dose delivered to the mucosa when applied extra-orally will not be the same and additional considerations are required [14]. The results of our simulations highlight many of these considerations. Perhaps the most important is the considerable degree of attenuation observed in the transmitted fluence rate. Despite an applied fluence rate of approximately 26 mW/cm^2^, the measured transmitted fluence rate ranged between 0.47 mW/cm^2^ and 2.38 mW/cm^2^, a roughly 10- to 50-fold reduction. This emphasizes that the same treatment protocols used intraorally cannot be used extra-orally and that an extraoral PBMT protocol will likely require higher power and longer wavelength for increased tissue penetration [14,16]. A second important consideration is the variation in transmitted fluence rate: a range of 0.47 mW/cm^2^ to 2.38 mW/cm^2^ indicates that the same applied dose of PBMT in two patients can transmit doses of considerable difference, roughly 5-fold in our limited sample size. This is, in part, due to variation in orofacial anatomy, both in total thickness and in tissue composition [14,17,18]. Given that it is not feasible to obtain MR imaging in all patients receiving PBMT, and given that the anatomy varies in unpredictable ways, a treatment protocol will likely have to aim to treat the “median” patient, akin to pharmacological agents utilizing a standard dose.

## 5. Conclusions

An in vivo validation study was performed to quantify the accuracy of Monte Carlo simulations in determining extraoral PBMT dosimetry. Mean in vivo transmission measurements made were within 12% of values determined from simulations. With the accuracy of simulations confirmed, Monte Carlo techniques can be utilized to determine the appropriate parameters of extraoral PBMT procedures for oral mucositis treatment.

## Figures and Tables

**Table 1:** Optical properties of cheek tissues for 850 nm light.

Structure	Absorption Coefficient, /mm	Reduced Scattering Coefficient, /mm
Skin	0.056	1.65
Fat	0.009	1.1
Muscle	0.035	0.65

**Table 2:** Summary of validation data.

Subject	Gender	Age	Skin Type	Anatomy of Right Cheek with Open Mouth Obtained from MRI			Measured Transmission, mW/cm^2^	Simulated Transmission, mW/cm^2^	% Difference
				Skin, mm	Fat, mm	Muscle, mm			
1	M	25	IV	2	3	7	0.47	0.41	12
2	F	57	VI	1	0	5	2.14	2.12	1
3	F	25	II	1	2	3	2.38	2.07	11
4	M	43	I	2	1	6	0.83	0.82	1

## Data Availability

Datasets related to this article can be obtained from the corresponding author.

## References

[1] Elting L.S., Cooksley C., Chambers M., Cantor S.B., Manzullo E., Rubenstein E.B. (2003). The burdens of cancer therapy: Clinical and economic outcomes of chemotherapy-induced mucositis. Cancer.

[2] Rubenstein E.B., Peterson D.E., Schubert M., Keefe D., McGuire D., Epstein J., Elting L.S., Fox P.C., Cooksley C., Sonis S.T. (2004). Clinical practice guidelines for the prevention and treatment of cancer therapy–induced oral and gastrointestinal mucositis. Cancer.

[3] Chaudhry H.M., Bruce A.J., Wolf R.C., Litzow M.R., Hogan W.J., Patnaik M.S., Kremers W.K., Phillips G.L., Hashmi S.K. (2016). The Incidence and Severity of Oral Mucositis among Allogeneic Hematopoietic Stem Cell Transplantation Patients: A Systematic Review. Biol. Blood Marrow Transplant..

[4] Sonis S.T. (2004). The pathobiology of mucositis. Nat. Rev. Cancer.

[5] Oberoi S., Zamperlini-Netto G., Beyene J., Treister N.S., Sung L. (2014). Effect of prophylactic low level laser therapy on oral mucositis: A systematic review and meta-analysis. PLoS ONE.

[6] Palma L.F., Gonnelli F.A.S., Marcucci M., Dias R.S., Giordani A.J., Segreto R.A., Segreto H.R.C. (2017). Impact of low-level laser therapy on hyposalivation, salivary pH, and quality of life in head and neck cancer patients post radiotherapy. Lasers Med. Sci..

[7] Paglioni M.D.P., Alves C.G.B., Fontes E.K., Lopes M.A., Ribeiro A.C.P., Brandão T.B., Migliorati C.A., Santos-Silva A.R. (2019). Is photobiomodulation therapy effective in reducing pain caused by toxicities related to head and neck cancer treatment? A systematic review. Support. Care Cancer.

[8] Zadik Y., Arany P.R., Fregnani E.R., Bossi P., Antunes H.S., Bensadoun R.-J., Gueiros L.A., Majorana A., Nair R.G., Elad V.R. (2019). Systematic review of photobiomodulation for the management of oral mucositis in cancer patients and clinical practice guidelines. Support. Care Cancer.

[9] Treister N.S., London W.B., Guo D., Malsch M., Verrill K., Brewer J., Margossian S., Duncan C. (2016). A feasibility study evaluating extraoral photobiomodulation therapy for prevention of mucositis in pediatric hematopoietic cell transplantation. Photomed. Laser Surg..

[10] Adnan A., Yaroslavsky A.N., Carroll J.D., Selting W., Juliano A.F., London W.B., Sonis S.T., Duncan C.N., Treister N.S. (2021). The Path to an Evidence-Based Treatment Protocol for Extraoral Photobiomodulation Therapy for the Prevention of Oral Mucositis. Front. Oral Health.

[11] Yaroslavsky A. (1999). Spectroscopic Investigations of Biological Tissues and Fluids. Ph.D. Thesis.

[12] Keijzer M., Jacques S.L., Prahl S.A., Welch A.J. (1989). Light distributions in artery tissue: Monte Carlo simulations for finite-diameter laser beams. Lasers Surg. Med..

[13] Tseng S.H., Bargo P., Durkin A., Kollias N. (2009). Chromophore concentrations, absorption and scattering properties of human skin in-vivo. Opt. Exp..

[14] Simpson C.R., Kohl M., Essenpries M., Cope M. (1998). Near-infrared optical properties of ex vivo human skin and subcutaneous tissues measured using the Monte Carlo inversion technique. Phys. Med. Biol..

[15] Hodgson B.D., Margolis D.M., Salzman D.E., Eastwood D., Tarima S., Williams L.D., Sande J.E., Vaughan W.P., Whelan H.T. (2012). Amelioration of oral mucositis pain by NASA near-infrared light-emitting diodes in bone marrow transplant patients. Support. Care Cancer.

[16] Chung H., Dai T., Sharma S.K., Huang Y.Y., Carroll J.D., Hamblin M.R. (2012). The nuts and bolts of low-level laser (light) therapy. Ann. Biomed. Eng..

[17] Chopra K., Calva D., Sosin M., Tadisina K.K., Banda A., De La Cruz C., Chaudhry M.R., Legesse T., Brachenberg C.B., Manson P.N. (2015). A comprehensive examination of topographic thickness of skin in the human face. Aesthetic Surg. J..

[18] Hwang K., Kim H., Kim D.J. (2016). Thickness of skin and subcutaneous tissue of the free flap donor sites: A histologic study. Microsurgery.

